# Low Intake of Zinc and Vitamin D Is Associated with High Blood Lead Level Proportion Amongst Male Workers with Lead Exposure

**DOI:** 10.3390/nu18111772

**Published:** 2026-05-30

**Authors:** Ade Mutiara, Diana Sunardi, Safarina G. Malik, Wawaimuli Arozal, Ninik Sukartini, Aria Kekalih, Nani C. Sudarsono, Dicky L. Tahapary, Stephan Boese O’Reilly, Muchtaruddin Mansyur

**Affiliations:** 1 Doctoral Program, Faculty of Medicine, Universitas Indonesia, Jakarta 10430, Indonesia; 2 Indonesia Medical Education and Research Institute (IMERI), Faculty of Medicine, Universitas Indonesia, Jakarta 10430, Indonesia; muchtaruddin.mansyur@ui.ac.id; 3 Department of Nutrition, Faculty of Medicine, Universitas Indonesia—Dr. Cipto Mangunkusumo General Hospital, Jakarta 10430, Indonesia; diana_sunardi@yahoo.com; 4 Genome Diversity and Disease Division, Mochtar Riady Institute for Nanotechnology, Universitas Pelita Harapan, Tangerang 15811, Indonesia; safarina.malik@mrinstitute.org; 5 Department of Pharmacology, Faculty of Medicine, Universitas Indonesia, Jakarta 10430, Indonesia; wawaimuli@gmail.com; 6 Department of Clinical Pathology, Faculty of Medicine, Universitas Indonesia—Dr. Cipto Mangunkusumo General Hospital, Jakarta 10430, Indonesia; ninik.sukartini@ui.ac.id; 7 Department of Community Medicine, Faculty of Medicine, Universitas Indonesia, Jakarta 10430, Indonesia; aria.kekalih@ui.ac.id (A.K.); nani.cahyani@ui.ac.id (N.C.S.); 8 Division of Endocrinology, Metabolism and Diabetes, Department of Internal Medicine, Faculty of Medicine, Universitas Indonesia—Dr. Cipto Mangunkusumo General Hospital, Jakarta 10430, Indonesia; dicky.tahapary@ui.ac.id; 9 Institute and Clinic for Occupational, Social and Environmental Medicine, LMU Medizin, LMU University Hospital, Ludwig-Maximilians-Universität München, Ziemssenstr. 5, D-80336 Munich, Germany; stephan.boeseoreilly@med.uni-muenchen.de

**Keywords:** blood lead levels, calcium intake, carbohydrate, Java workers, nutrition intake, occupational exposure, protein, vitamin D, waist-to-height ratio, zinc

## Abstract

**Background/Objectives:** Nutritional intake plays an important role in modulating lead absorption and toxicity. In addition to micronutrient status, emerging evidence suggests that body fat distribution may influence heavy metal toxicokinetics, yet this aspect remains poorly explored in occupational settings. This study aimed to investigate the associations of dietary intake of zinc, calcium, vitamin D, and protein, as well as anthropometric indicators, with blood lead levels (BLLs) among lead-exposed male workers. **Methods:** A cross-sectional study was conducted involving 144 male workers from five areas with varying degrees of environmental lead contamination in Java, Indonesia. Nutrient intake was assessed using a semi-quantitative food frequency questionnaire (SQ-FFQ). Anthropometric measurements included body mass index (BMI) and waist-to-height ratio (WHtR). BLLs were measured using inductively coupled plasma mass spectrometry (ICP-MS). Bivariate and multivariate analyses were performed to identify independent predictors of elevated BLLs. **Results:** The median BLL was 6.8 µg/dL (Q1–Q3: 4.75–13.08), and 32% of participants had BLLs above 10 µg/dL. BLLs differed significantly across exposure areas (*p* < 0.001). In bivariate analysis, WHtR, protein intake, zinc intake, and vitamin D intake were significantly associated with BLLs. Workers with higher WHtR had a greater proportion of elevated BLLs (*p* = 0.023), whereas BMI was not associated. In multivariate analysis, low zinc intake (*p* = 0.031) and low vitamin D intake (*p* = 0.021) remained significant predictors of high blood lead levels. **Conclusions:** Environmental exposure remains the main determinant of BLLs, while low intake of zinc and vitamin D increases the risk of high blood lead levels. Central adiposity, reflected by WHtR, may represent a potential anthropometric marker of lead burden, suggesting a potential role of body fat distribution in lead toxicokinetics that warrants further investigation.

## 1. Introduction

Lead exposure remains a significant yet often underestimated global health concern, particularly in low- and middle-income countries where industrial use of lead persists, and regulatory enforcement is limited [1]. Lead is a pervasive environmental and occupational toxicant, with no safe level of exposure identified. Even at low blood lead concentrations, adverse health effects have been documented, including neurocognitive deficits, metabolic disturbances, renal impairment, and bone demineralization [2].

Despite well-established evidence of its toxicity, lead continues to be utilized in industrial processes such as battery manufacturing, metal recycling, pigment production, and soldering, especially in low- and middle-income countries [3]. Workers in such industries are especially vulnerable to chronic lead exposure due to prolonged occupational contact, primarily through inhalation of lead dust and fumes or inadvertent ingestion from hand-to-mouth contact in contaminated environments. Chronic lead exposure has been associated with a wide range of adverse health effects, including neurocognitive deficits, cardiovascular disease, renal dysfunction, and metabolic disorders such as insulin resistance and type 2 diabetes [4,5]. Unlike an acute toxicant, lead accumulates gradually in the body, and its harmful effects may not become apparent until significant damage has occurred.

Nutritional status plays a critical role as a modifiable factor influencing both the absorption and toxicity of lead. Several nutrients, including calcium, zinc, vitamin D, and protein, play essential roles in maintaining homeostatic mechanisms that may either attenuate or exacerbate lead absorption [6]. Calcium competes with lead for intestinal absorption and bone deposition. Zinc is an essential trace element widely distributed in eukaryotic cells, where it participates in hundreds of proteins and enzymes as a catalytic and structural cofactor and functions as a key regulatory ion in cellular growth and differentiation [7]. Zinc also induces metallothionein and modulates oxidative stress responses, while vitamin D influences bone turnover, thereby regulating the mobilization of lead from skeletal stores [8]. Protein intake, while essential for growth and repair, may have complex interactions with lead exposure depending on dietary sources, as some protein-rich foods are also significant routes of dietary lead exposure [9].

Micronutrient deficiencies continue to be a widespread public health concern in low- and middle-income countries, especially among at-risk populations. These deficiencies are primarily driven by factors such as poverty, insufficient dietary variety, inadequate nutrition programs, poor delivery of health services, and barriers to accessing healthcare. The most common micronutrient deficiencies in people are iron, vitamins A and D, iodine, and zinc [10]. These nutritional deficiencies may amplify the toxic effects of occupational lead exposure. Previous studies have shown inconsistent findings regarding nutrient–lead interactions. Some have demonstrated an inverse association between dietary calcium or zinc and BLLs [2,11,12], whereas others have found minimal or no correlation after adjusting for confounding factors such as age, smoking, and work duration [13].

Beyond micronutrients, an emerging but underexplored dimension in lead toxicology is the role of body composition, particularly fat distribution. While body mass index (BMI) has traditionally been used as an indicator of nutritional status, it does not capture central adiposity, which is more closely linked to metabolic dysfunction and inflammation. The waist-to-height ratio (WHtR) has been proposed as a simple indicator of central fat accumulation and cardiometabolic risk. Recent population-based studies suggest that central obesity may be associated with higher blood concentrations of heavy metals, including lead, but this relationship has rarely been examined in occupational cohorts [14,15].

To address these knowledge gaps, the present study aims to investigate the associations of dietary intake of protein, calcium, zinc, and vitamin D, as well as anthropometric indicators, with blood lead levels in Indonesian lead-exposed industrial workers. By clarifying these relationships, the study seeks to provide evidence for nutritional strategies as part of lead exposure management and occupational health interventions to better understand the potential protective role of nutrition in lead toxicity.

## 2. Materials and Methods

### 2.1. Study Population and Design

A cross-sectional study was conducted to examine the associations of the nutrient intake of protein, zinc, calcium, and vitamin D with BLLs in lead-exposed workers. All participants provided informed consent prior to enrollment. Participants were selected from areas identified as having significant lead exposure, based on the presence of used lead–acid battery (ULAB) recycling activities and soil lead concentration measurements. Eligible participants were male workers aged 18–64 years [16].

### 2.2. Study Area

**Figure 1:**
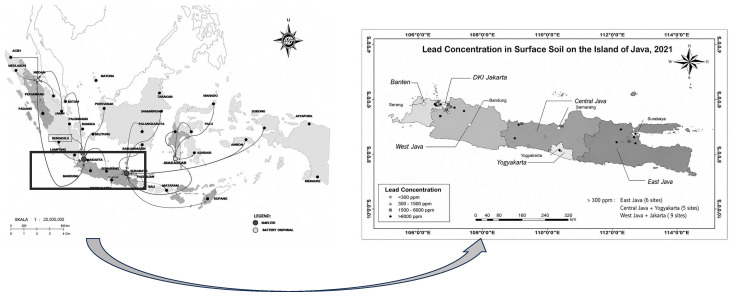
Distribution of lead battery trading in Indonesia (lead exposure and Indonesian children’s health in Java Island)—left map: Lead soil concentration in Java Island, 2021. Source: The data displayed in the map are based on a 2021–2022 assessment of lead-contaminated sites conducted under the Protecting Every Child’s Potential (PECP) initiative (right map) [17].

The study area was selected based on soil lead concentrations measured across five locations representing varying degrees of environmental contamination [16]. Significant disparities in soil lead concentrations were observed; median (Q1–Q3) values were 6581.7 ppm (2432.6–16,647.1) in high-exposure areas, 1132.4 ppm (214.0–3341.0) in medium-exposure areas, and 253.5 ppm (158.8–417.1) in control areas. Four sites exhibited elevated soil lead levels, whereas one site demonstrated undetectable or minimal concentrations. The high-exposure sites comprised Pesarean Village (Tegal Regency, Central Java) and Cinangka Village (Bogor Regency, West Java), both characterized by high soil lead concentrations and occupational activities involving lead. Medium exposure was determined for Kadu Jaya Village (Tangerang Regency, Banten), an industrial area hosting a used-battery recycling industry, and Jalan Demak (Surabaya City, East Java), known for extensive used-battery trading activities. To ensure the inclusion of participants representing low background exposure levels, Cinangneng Village (Bogor Regency, West Java) was incorporated as a comparative reference site. The map of the sites in this study is provided in Figure 1.

### 2.3. Data Collection

Data collection was conducted using purposive sampling, which started by collecting the data of potential subjects exposed to lead from subdistrict public health centers and village offices. This cross-sectional study was reviewed and approved by the Ethics Committee of the Faculty of Medicine, Universitas Indonesia—Cipto Mangunkusumo Hospital (No. ND-866/UN2.F1/ETIK/PPM.00.02/2023) on 20 March 2023. All participants provided written informed consent. Prospective participants were evaluated based on the established inclusion and exclusion criteria as follows: male, 18–60 years old, current residence or workplace in the selected community, and willingness to participate in this study by signing written informed consent. The exclusion criterion was the use of vitamin D supplementation. Sociodemographic data were collected through structured interviews using a standardized questionnaire. Information gathered included age, education level, work duration, sun exposure, and job position. Anthropometric measurements were collected using standardized and calibrated equipment. Body weight was measured with a digital scale to the nearest 0.1 kg, and height was assessed with a stadiometer to the nearest 0.1 cm. Waist circumference was measured at the midpoint between the lower margin of the last palpable rib and the iliac crest using a non-stretchable measuring tape, recorded to the nearest 0.1 cm. Nutrient intake data, including protein, zinc, calcium, and vitamin D, were assessed using a semi-quantitative food frequency questionnaire (SQ-FFQ) adapted from instruments validated in the Indonesian population [18]. The SQ-FFQ consisted of approximately 120 food items commonly consumed in the study area. Participants were asked to report their usual frequency of consumption (per day, week, or month) and portion sizes, supported by visual aids (household measures and calibrated utensils) to enhance accuracy. A trained data collector interviewed participants to record the intake of major food groups, portion sizes, and the frequency of consumption over the prior month (FFQ) or prior 24 h (recall). Nutrient intakes (protein, calcium, zinc, and vitamin D) were estimated using the Indonesian Food Composition Table and NutriSurvey software [19]. Nutrient intake data were grouped into two categories based on their median values and interpreted in terms of RDA percentages.

### 2.4. Sample Collection

Venous blood samples were collected in trace-element-free EDTA tubes for the determination of blood lead levels. Analyses were performed at a certified laboratory using inductively coupled plasma mass spectrometry (ICP-MS) (Agilent 7700, Agilent Technologies, Santa Clara, CA, USA) [20]. Analytical quality assurance and quality control (QA/QC) procedures included the use of reagent blanks, duplicate samples, and certified reference materials to verify accuracy and precision. Recovery rates were within acceptable limits, and calibration linearity was confirmed across the analytical range. Quality assurance and control procedures were applied throughout the analysis to ensure the accuracy and precision of the results.

The limit of detection (LOD) for lead ranged from 0.02 to 0.05 µg/dL, reflecting minor variation across analytical runs and calibration batches. All results were expressed in micrograms per deciliter (µg/dL).

### 2.5. Statistical Analysis

Descriptive statistics summarized participants’ sociodemographic and anthropometric characteristics. Categorical variables were presented as frequencies and percentages, whereas continuous variables were tested for normality using the Kolmogorov–Smirnov test and expressed as mean ± SD or median (Q1–Q3), as appropriate. Associations between nutrient intakes (protein, calcium, zinc, vitamin D) and blood lead levels were examined using independent *t*-tests or Mann–Whitney U tests. Considering the modest sample size, a stricter significance level (*p* < 0.01) for variable selection was adopted in the multiple logistic regression model to minimize type I error and enhance the reliability of the associations in identifying independent predictors of blood lead levels [21]. Results were reported as odds ratios with 95% confidence intervals, and *p* < 0.05 was considered statistically significant.

## 3. Results

### 3.1. Study Subject Characteristics

A total of 144 subjects were eligible for the study. Table 1 presents the characteristics of the subjects, who had a median age of 39 years, with a slightly higher proportion of individuals aged <40 years (55%). A total of 60% of subjects had a BMI below 23 kg/m^2^, indicating a predominantly normal-weight population. Smoking was dominant in the study population, with 92% of the subjects being smokers. Similarly, 58% of subjects had a waist-to-height ratio below 0.5. Blood lead levels (BLLs) were detected in all participants, ranging from 1.17 to 58.8 µg/dL, with 38% of subjects residing in high-exposure areas.

Table 2 shows nutrient intake over the prior month. The median protein intake was 54.2 g, corresponding to approximately 80% of the recommended dietary allowance (RDA). Approximately 51% of participants consumed less than 54.2 g of protein, which is below the recommended daily intake of 65 g. For zinc intake, subjects were evenly distributed between intake categories with a median of 5.9 mg of zinc. As for calcium and vitamin D, half of the participants (51%) consumed more than 380 mg of calcium and 1.2 µg of vitamin D (51%), which is also below the recommended daily intake of 1000 mg for calcium and 15 µg for vitamin D.

### 3.2. Association Between Variables

Participants with BLLs ≥ 10 µg/dL exhibited notable differences across several variables. Although BMI categories were not significantly associated with elevated blood lead levels (BLLs) (*p* = 0.278), the proportion of subjects with elevated BLLs tended to be higher among individuals with increased waist-to-height ratios compared with those with normal ratios (*p* = 0.023). Smoking was not associated with BLLs (*p* = 0.110). A notable difference in proportions was also observed across lead exposure areas, where subjects residing in high-exposure areas showed a substantially higher proportion of elevated BLLs compared with those from medium- and low-exposure areas (*p* < 0.001). Regarding nutrient intake, the proportion of participants with elevated BLLs differed significantly according to the intake of protein, zinc, and vitamin D (*p* = 0.045, *p* = 0.005, and *p* = 0.009, respectively). Variables with *p*-values <0.01 in the bivariate analysis were subsequently considered as candidate variables for inclusion in the multivariate model. Completed result on the bivariate analysis is provided in File S1 (Table S1).

**Figure 2:**
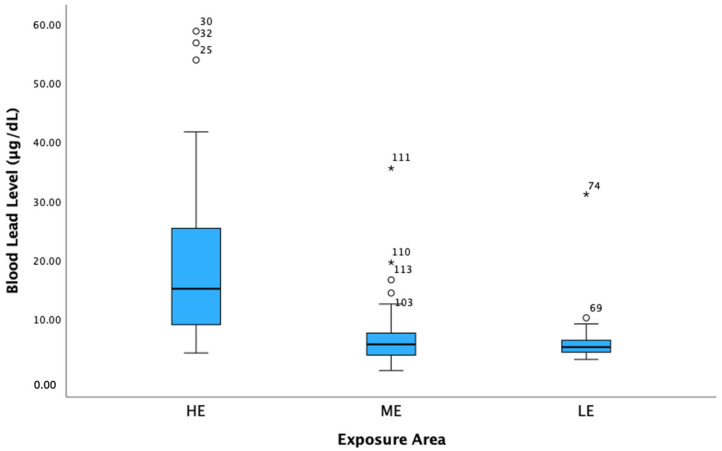
Distribution of blood lead levels across the exposure areas. HE: high exposure (n: 56); ME: medium exposure (n: 41); LE: low exposure (n: 47). * outliers.

Figure 2 shows the difference in the proportion of BLL within the three exposure areas, which showed that the median BLL in the high-exposure (HE) area was 15 µg/dL, while the medium-exposure (ME) and low-exposure (LE) areas showed a lower BLL value, with 5.6 µg/dL and 5.2 µg/dL.

### 3.3. Factors Associated with Blood Lead Levels

Multivariate logistic regression analysis using the enter method was performed to identify independent predictors of elevated BLLs. The stricter significance threshold (*p* < 0.01) applied during variable selection resulted in only two variables meeting the criteria for inclusion in the multivariate logistic regression model. In the final model, low zinc intake and low vitamin D showed a significant association with elevated BLLs. Participants with low zinc and vitamin D intakes had higher odds of elevated BLLs compared with those with higher intakes (aOR = 2.41; 95% CI: 1.15–5.07, *p* = 0.021 and aOR = 2.27; 95% CI: 1.08–4.76, *p* = 0.030) (Table 3).

### 3.4. Nutrient Factors Associated with High BLL Based on the Exposure Area

Table 4 shows the nutrient intake that contributed to the high BLLs based on the area of exposure. The analysis using the Mantel–Haenszel test showed that zinc was a significant factor in the high-exposure area (*p* = 0.040) but was not statistically significant in the medium- and low-exposure areas. Meanwhile, vitamin D intake did not show statistically significant differences among the exposure area groups. For the total study population across all exposure areas, both low zinc intake and low vitamin D intake were significant factors associated with high BLLs (*p* = 0.004 and *p* = 0.005).

## 4. Discussion

In this population of male industrial workers, the median BLLs of 6.8 µg/dL and nearly one-third of subjects exceeding 10 µg/dL indicate a substantial burden of ongoing occupational exposure. These levels are comparable to those reported among informal battery recyclers and metal-handling workers in other developing regions [22]. Such exposure patterns reveal the persistent challenge of regulating lead in small-scale industries, where occupational hygiene and enforcement remain limited. The marked differences in BLLs across exposure zones in this study support the conclusion that environmental contamination remains the dominant driver of internal lead burden. Specifically, 83% of workers in the high-exposure area had BLLs >10 µg/dL, compared to 13% and 4% in the medium- and low-exposure areas. Nearly all participants may be exposed to high environmental lead; consequently, the incremental impact of smoking was less statistically significant in comparison.

A key finding of the present study is that the proportion of high BLLs is significantly associated with low zinc and vitamin D intakes after multivariate adjustment. This observation is consistent with studies showing that zinc deficiency is linked to increased expression of the 100 kDa cyclic adenosine monophosphate (cAMP) responsive element modulator (CREM) transcription factor, which suppresses interleukin-2 (IL-2) production. Previous studies indicate that zinc may help attenuate the toxic effects of lead [11,23]. The findings also showed that lead exposure elevated 100 kDa CREM expression, with a more pronounced increase under zinc-deficient conditions. Accordingly, IL-2 production was significantly reduced in cells subjected to both lead exposure and zinc deficiency compared to those exposed to lead alone.

Based on the exposure area, this observation is consistent with mechanistic and epidemiological evidence showing that zinc plays a protective role against lead absorption and toxicity. Besides the above mechanism, another study showed that zinc and lead share common intestinal transport pathways, notably divalent metal transporter 1 (DMT1), and compete for binding to metallothionein, a metal-binding protein involved in detoxification [8,11]. Adequate zinc intake upregulates metallothionein expression, thereby sequestering lead in the intestinal mucosa and promoting fecal excretion rather than systemic circulation. A cross-sectional study among industrial workers reported that higher blood zinc concentrations were associated with lower BLLs and improved antioxidant enzyme activity, supporting a biologically plausible protective mechanism [8,24]. Given that mild zinc deficiency is still common among Indonesian male adults [25] ensuring sufficient zinc consumption could represent a practical, low-cost intervention to reduce lead absorption in high-risk occupations. The same study on zinc deficiency shows that zinc supplementation counteracted these effects, preventing CREM 100 kDa overexpression and restoring IL-2 levels. In conclusion, we identified CREM 100 kDa as a potential molecular mechanism behind the lead-induced IL-2 decrease in Jurkat T cells, with zinc deficiency exacerbating this effect [23].

This study showed that low intake of vitamin D is statistically significantly related to the odds of elevated BLLs. While zinc exhibits a notably different protective effect, especially in high-exposure areas, vitamin D in this study did not show any difference in its protective factor based on the exposure area. However, in the analysis of the total population, vitamin D levels showed a protective role without observing the exposure area. This protective factor is consistent with a study showing that vitamin D may protect against blood lead exposure from the bone reservoir by decreasing bone turnover [26], although, on the other hand, one study showed that high blood lead levels (BLLs) may have a significant impact on circulating vitamin D3 levels and bone resorption in workers occupationally exposed in smelters in India [27]. One other study supports the role of vitamin D in modulating the expression of genes involved in lead transport and excretion, which may also contribute to its protective effect [28]. In Zhang’s study, a sufficient level of vitamin D in the blood was found to play a protective role in reducing the bioavailability of lead, and a threshold effect was observed at a vitamin D level of 38.679 ng/mL, suggesting an optimal concentration range for reducing blood lead levels (BLLs). A cross-sectional study in Indonesia amongst the pre-frail adult population showed that only 11.6% of the participants had normal vitamin D levels [29]. With a median of only 8% of the RDA, this might be a possible factor why vitamin D did not show any difference amongst different areas of exposure in our study. However, amidst the low vitamin D intake population, this study showed that higher vitamin D intake contributed to lower BLLs. Another study also mentioned that vitamin D plays multiple roles, particularly in calcium metabolism and bone health. Lead exposure can impair the renal hydroxylation of 25(OH)D, while insufficient vitamin D levels lead to elevated parathyroid hormone (PTH), thereby reducing calcium absorption in the body [30].

Calcium intake has been shown to influence lead toxicokinetics primarily at the level of gastrointestinal absorption rather than circulating blood lead levels. Experimental and observational evidence indicate that calcium and lead compete for similar absorption pathways in the intestine, such that inadequate calcium intake enhances lead absorption and retention, particularly among vulnerable populations such as children [31]. This is supported by the Agency for Toxic Substances and Disease Registry [4]. This competitive interaction suggests that calcium plays a protective role at the entry point of exposure, limiting the amount of lead that enters systemic circulation. However, BLL is widely recognized as a biomarker of recent exposure rather than nutritional status. According to the Centers for Disease Control and Prevention (CDC, 2022), BLL reflects ongoing or recent environmental exposure, while earlier studies have demonstrated that environmental and occupational sources account for the majority of variability in BLL [32]. Consequently, the relationship between calcium intake and BLL may appear weak or inconsistent in epidemiological studies, as nutritional factors exert their influence primarily during absorption, whereas measured BLL is more strongly determined by the intensity and duration of external exposure. The reason for choosing male subjects for this study is that gender-related differences in bone metabolism contribute to variations in lead kinetics. In postmenopausal women, estrogen deficiency increases bone resorption, promoting the release of stored lead into circulation. This endogenous source, together with environmental exposure, may elevate blood lead levels [33].

The observed association between WHtR and elevated BLLs suggests a potential role of central adiposity in lead toxicokinetics. Adipose tissue has been implicated in the storage and redistribution of lipophilic and certain metal compounds, while central obesity is associated with chronic low-grade inflammation, which may influence metal metabolism and oxidative stress pathways. Although evidence remains limited, emerging studies suggest that central fat accumulation may modify the internal distribution and biological effects of heavy metals, including lead [34].

This study highlights the critical role of nutrient adequacy, particularly vitamin D and zinc, in modulating the biological impact of lead exposure among workers. From an implementation perspective, it is essential to strengthen community awareness of the importance of adequate micronutrient intake, especially in high-risk populations. At the policy level, coordinated advocacy is needed to support the integration of environmental exposure control with nutritional interventions to target the insufficiency of micronutrients, specifically vitamin D and zinc. This includes the development of national and local strategies that mandate surveillance programs and promote preventive approaches such as food-based interventions and targeted supplementation. In parallel, occupational health services should be enhanced to incorporate routine surveillance of vitamin D and zinc status as part of comprehensive health monitoring in lead-exposed communities. Such a multidimensional approach is crucial to mitigate the health risks associated with lead exposure and to address the widespread insufficiency of key micronutrients in vulnerable populations.

### Study Limitation

This study is limited by its cross-sectional design, which precludes causal inference. Additionally, key determinants of vitamin D status, including serum 25(OH)D levels and sun exposure, were not measured and may have confounded the observed associations. As the study focused on dietary intake, these factors were beyond its scope; therefore, the findings should be interpreted as associative rather than causal. Future studies incorporating biochemical assessments and environmental exposure variables are warranted to provide a more comprehensive understanding of these relationships.

## 5. Conclusions

The present study suggests that environmental exposure area and low zinc and vitamin D intakes are the main factors associated with elevated blood lead levels among lead-exposed workers in Java, Indonesia. Furthermore, the potential observed association between waist-to-height ratio and BLLs indicates that central adiposity may play a role in lead toxicokinetics, highlighting the need for further investigations. In conclusion, integrating micronutrient adequacy, particularly vitamin D and zinc, into surveillance and policy frameworks is essential to mitigate the health impact of lead exposure. A coordinated approach combining community education, routine monitoring, and targeted nutritional interventions offers a practical and scalable strategy for high-risk populations.

## Figures and Tables

**Table 1:** Characteristics of study subjects.

Variables	n (%)	Median (Q1–Q3)
Age (year)		39 (20–59)
≥40 <40	63 (45) 78 (55)	
BMI (kg/m^2^)		21.6 (15.2–34.2)
≥23 <23	57 (40) 87 (60)	
Waist-to-height ratio		0.48 (0.37–0.67)
≥0.5 <0.5	61 (42) 83 (58)	
Smoking habit		
Yes	133 (92)	
No	31 (8)	
Blood lead level (µg/dL)		6.8 (1.2–58.8)
≥10 <10	45 (32) 96 (68)	
Exposure area		
High	56 (39)	
Medium	41 (29)	
Low	47 (32)	

**Table 2:** Nutrient intake proportion distribution-based median cut-off.

Nutrient	n (%)	Median (Q1–Q3)
Protein intake (gram)/RDA		54.2 (17–169)
<54.2/<80% ≥54.2/≥80%	73 (51) 71 (49)	
Zinc intake (mg)/RDA		5.9 (2.1–20.9)
<5.9/<54% ≥5.9/≥54%	72 (50) 72 (50)	
Calcium intake (mg)/RDA		380 (51–1426)
<380/<37% ≥380/≥37%	73 (51) 71 (49)	
Vitamin D intake (mcg)/RDA		1.2 (0–24.7)
<1.2/<8% ≥1.2/≥8%	74 (51) 70 (49)	

RDA = recommended dietary allowance.

**Table 3:** Multivariate logistic regression analysis of factors associated with elevated BLLs.

	aOR *	95% CI	*p*-Value
Zinc intake (mg)	2.41	1.15–5.07	0.021
Vitamin D intake (mcg)	2.27	1.08–4.76	0.030

* Variables adjusted for each other. Variable selection was based on *p* < 0.01 from File S1 (Table S1).

**Table 4:** Nutrient intake associated with elevated BLLs based on exposure area.

Exposure Area	Category	BLL (µg/dL)		*p*-Value
		≥10 n;%	<10 n;%	
	**Zinc Intake (mg)**			
High	<5.9	27;68	6;38	0.040 *
	≥5.9	13;32	10;62	
Medium	<5.9	3;50%	17;49	0.645
	≥5.9	3;50%	18;51	
Low	<5.9	2;100	17;38	0.158
	≥5.9	0;0	28;62	
Total	<5.9	32;67	40;42	0.004 *
	≥5.9	16;33	56;58	
	**Vitamin D Intake (mcg)**			
High	<1.2	29;73	9;56	0.194
	≥1.2	11;27	7;44	
Medium	<1.2	1;17	18;51	0.128
	≥1.2	5;83	17;49	
Low	<1.2	1;50	12;27	0.481
	≥1.2	1;50	33;73	
Total	<1.2	31;65	39;41	0.005 *
	≥1.2	17;35	57;59	

* *p* < 0.05.

## Data Availability

The raw data supporting the conclusions of this article will be made available by the authors on request.

## Supplementary Materials

The following supporting information can be downloaded at https://www.mdpi.com/article/10.3390/nu18111772/s1, File S1: Table S1. Association analysis between anthropometric measurements, nutrient intake, exposure area, and BLLs. File S2: Semi-quantitative FFQ Questionnaire Form [18]; File S3: Stratified Data by Area.

## Abbreviations

The following abbreviations are used in this manuscript:

BLLs	Blood lead levels
FFQ	Food frequency questionnaire
BMI	Body mass index
WHtR	Waist-to-height ratio
ICP-MS	Inductively coupled plasma mass spectrometry
ULAB	Used lead–acid battery
LOD	Limit of detection
AAS	Atomic absorption spectrometry
RDA	Recommended dietary allowance
