# Supplementary material for: Low Intake of Zinc and Vitamin D Is Associated with High Blood Lead Level Proportion Amongst Male Workers with Lead Exposure
Source: Nutrients. 2026 May 30;18(11):1772. doi: 10.3390/nu18111772 (PMC13259502; doi:10.3390/nu18111772)
Supplement: Supplementary file 1 [file nutrients-18-01772-s001.zip › nutrients-4262075-supplementary/File S1.pdf]

Table S1. Association analysis between anthropometric measurements, nutrient intake, exposure area, and BLLs.

Variables	BLL (μg/dL)		*p*-Value
	≥10	<10	
BMI (kg/m²)			0.278
≥23	16 (33%)	41 (43%)	
<23	32 (67%)	55 (57%)	
Waist-to-height ratio			0.023
≥0.5	14 (29%)	47 (49%)	
<0.5	34 (71%)	49 (51%)	
Smoking habit			0.110
Yes	41 (36)	72 (64)	
No	7 (23)	24 (77)	
Protein intake (gram)			0.045
<54.2	30 (63%)	43 (45%)	
≥54.2	18 (37%)	53 (55%)	
Zinc intake (mg)			0.005 *
<5.9	32 (67%)	40 (42%)	
≥5.9	16 (33%)	56 (58%)	
Calcium intake (mg)			0.099
<380	29 (60%)	44 (46%)	
≥380	19 (40%)	52 (54%)	
Vitamin D intake (mcg)			0.009 *
<1.2	32 (67%)	42 (44%)	
≥1.2	16 (33%)	54 (56%)	
Exposure area			<0.001
High	40 (83%)	16 (17%)	
Medium	6 (13%)	35 (37%)	
Low	2 (4%)	45 (47%)	

* $p < 0.01$.
