# Supplementary material for: Low Intake of Zinc and Vitamin D Is Associated with High Blood Lead Level Proportion Amongst Male Workers with Lead Exposure
Source: Nutrients. 2026 May 30;18(11):1772. doi: 10.3390/nu18111772 (PMC13259502; doi:10.3390/nu18111772)
Supplement: Supplementary file 1 [file nutrients-18-01772-s001.zip › nutrients-4262075-supplementary/File S2_SQ-FFQ form.pdf]

Supplement S2: Semi-quantitative FFQ Questionnaire Form

No. Sample :

Name :

Age :

Gende :

Education :

Occupation :

Date of Interview :

Enumerator :

Handphone :

Food Material	How many times to consume per....							Portion per consumption		Most often cooked in this way...
	>1x /day	1x /day	4-6x /week	3x /week	<3x /week	Every other week	Never	Household unit	Gram	
Grain and tuber groups										
Rice noodles										
Black sticky rice										
White sticky rice										
White Rice										
Brown rice										
Oatmeal										
Yellow corn										

White cassava										
Yellow cassava										
Potatoe										
Macaroni										
Taro										
Rice flour										
Cornstarch										
Sago flour										
Flour										
Red sweet potato										
White sweet potato										
Nut Group										
Emping										
Jengkol										
Chickpeas										
Bogor Nuts										
Green beans										
Soybeans										
Red beans										
Jack beans										
peeled peanuts										
Soy sauce										
Sunflower seed										
Green coconut, water										
Green coconut, flesh										

powdered soybean juice										
Coconut milk										
Soymilk										
soy tempeh										
Chinese stinky bean										
Fermented soy										
Tofu										
Sesame seed										
Vegetable										
Spinach										
Red spinach										
Onion										
Jicama										
Beets										
Beans										
Broccoli										
Green pepper										
Red pepper										
Cayenne pepper										
Leek										
Basil leave										
Melinjo leaves										
Sweet potato leaves										
Fern Leaves										
Pohpohan leaves										

Papaya leaves										
Cassava leaves										
green corn										
Button mushrooms										
Ear mushroom										
Shitake mushroom										
Oyster mushroom										
Banana heart										
Gambas										
Genjer										
Kailan										
Kangkung										
Katuk										
green Kapri										
Kapri beans										
Long beans										
Cucumber										
Kecipir										
Cauliflower										
White cabbage										
Red cabbage										
Chives										
Chayote										
Water gourd										
Turnip										
Green jackfruit										
green papaya										
Pare										
Pecay										

Sugarcane										
Bamboo shoots										
Chinese cabbage										
Mustard greens										
Watercress										
Celery										
Mung bean sprouts										
ripe tomatoes										
Green tomatoes										
Carrot										
Eggplant										
Lettuce										
Gambas										
Fruits										
Avocado										
Apple										
Strawberry										
Starfruit										
Jacima										
Cempedak										
Duku										
Durian										
Guava										
Water apple										
Pomelo										
Orange										
Lemon										

Kedondong										
Persimmon										
Kiwi										
Mango gadung										
Mango golek										
Mango harum manis										
Mango indramayu										
Green mango										
Mangosteen										
Jackfruit										
Pinapple										
Papaya										
Banana										
Rambutan										
Sapodilla										
Snakefruit										
Wartermelon										
Soursops										
Srikaya										
Fats and Oils										
Margarine										

Others										
Pudding										
Dodol										
Palm sugar										
Spy Sauce										
Coffee										
Sugar										
Ginger										
Honey										
Tomato sauce										
Drinks										
Ale-ale										
Buavita										
Fruitamin										
Marjan Syrup										
ABC syrup										
Lasegar										
Happy jus										
Frutang										
Jasjus										
Mr. Jussie										
Minute maid pulppy										
Mizone										
Sari asem										

Sari kacang ijo										
Floridina										
Nutri sari										
You C1000 (isotonik)										
Pocari sweat										
Kratindaeng										
Redbull										
Hemaviton energy										
Hemaviton C1000										
Levite jeruk										
Q-guava										
Lemon water										
Jungle juice										
Orange water										
v-soy										
Soya milk										
Sirup pohon pinang										
Sunkist										
Sunquick										

1. Apakah Anda mengkonsumsi suplemen zat besi, vitamin A, vitamin B12 dan vitamin C ?
   a. Yes (If yes, please fill in the table below.)
   b. b. No (If No, ignore))

Merk Suplemen	Dosis	**How many times to consume per....**						
		> 1x/day	1x/day	3-6x /week	1-2x /mweek	Every other week	Once a month	Never
You C1000								
Hemaviton C1000								
Alfalfa								
Nervita								
Acidophilus								
Biotamix								
Maxvita								
Sangobion								
vermia								
Sakatonik								
Neurobion								

c. 2. Are you currently taking any medication/have you taken any medication in the last month?

d. Yes (If yes, please fill in the table below.)

e. b. No (If No, ignore))

Nama obat	Ya	Tidak
Aspirin		
Ranitidin		
Antasid		
Omeprazol		
Lansoprazol		
Kloramfenikol		
Tetrasiklin		
Metformin		
Prednisolon		
Deksametason		
Alendronat		
Etidronat		
Risedronat		
Kolkisin		
Alopurinol		
Kolestiramin		
Interleukin 2		
Pemetreksed		
