# Supplementary material for: Low Intake of Zinc and Vitamin D Is Associated with High Blood Lead Level Proportion Amongst Male Workers with Lead Exposure
Source: Nutrients. 2026 May 30;18(11):1772. doi: 10.3390/nu18111772 (PMC13259502; doi:10.3390/nu18111772)
Supplement: Supplementary file 1 [file nutrients-18-01772-s001.zip › nutrients-4262075-supplementary/File S3_Stratified Data by Area.pdf]

Table S3. Characteristic Subject & Nutrition Intake of Subject by Exposure Area

Variables	High Exposure (HE)		Medium Exposure (ME)		Low Exposure (LE)	
	N(%)	Median (Q1-Q3)	N(%)	Median (Q1-Q3)	N(%)	Median (Q1-Q3)
**Characteristic**						
Age (year)		40 (22-59)*		39 (20-58)*		39 (21-55)*
≥40	24 (43)		18 (44)		22 (49)	
<40	32 (57)		23 (56)		25 (51)	
BMI (kg/m^2)		21.3 (19.0-24.1)		23.2 (19.6-25.7)		21.4 (20.5-25.6)
≥23	19 (34)		21 (51)		17 (36)	
<23	37 (66)		20 (49)		30 (64)	
Waist to Height Ratio		0.46 (0.43-0.50)		0.51 (0.45-0.54)		0.49 (0.43-0.55)
≥0,5	16 (29)		22 (54)		23 (49)	
<0,5	40 (71)		19 (46)		24 (51)	
Smoking Habit						
Yes	47 (84)		30 (73)		36 (77)	
No	9 (16)		11 (27)		11 (23)	
Blood Lead Level (μg/dL)		15.1 (8.96-25.60)		5.9 (3.75-7.57)		5.2 (4.28-6.33)
≥10	40 (71)		6 (15)		2 (4)	
<10	16 (29)		35 (85)		45 (96)	
**Nutrition Intake**						
Protein intake (gram)/RDA		48.2 (34.6-65.8)		53.8 (42.8-68.6)		75.4 (42.3-105.9)
<54.2/ <80%	34 (61)		22 (54)		17 (37)	
≥54.2/ ≥80%	22 (39)		19 (46)		30 (63)	
Zinc intake (mg)/RDA		5.4 (4.2-7.2)		6 (4.7-7.0)		7.5 (4.4-11.2)
<5.9/ <54%	33 (59)		20 (49)		19 (40)	

≥5.9/ ≥54%	23 (41)		21(51)		28 (60)	
Calcium intake (mg)/RDA		302.6 (218.6-468.8)		341.4 (232.9-453.6)		515.8 (281.5-744.1)
<379.7 /<37%	31 (56)		25 (61)		16 (34)	
≥379.7 /≥37%	25 (44)		16 (39)		31 (66)	
Vitamin D intake (mcg)/RDA		0.8 (0.4-1.6)		1.2 (0.5-2.5)		1.9 (0.6-3.5)
<1.2/<8%	39 (70)		21(51)		14 (30)	
≥1.2/≥8%	17 (30)		20 (49)		33 (70)	

*mean
